# Crowded skies: Conflicts between expanding goose populations and aviation safety

**DOI:** 10.1007/s13280-017-0901-2

**Published:** 2017-02-18

**Authors:** David R. Bradbeer, Camilla Rosenquist, Thomas Kjær Christensen, Anthony D. Fox

**Affiliations:** 1 Airside Operations, Vancouver Airport Authority, PO Box 23750, Airport Postal Outlet, Richmond, BC V7B 1Y7 Canada; 2 Københavns Lufthavne A/S, Lufthavnsboulevarden 6, Postboks 74, 2770, Kastrup Denmark; 3 Department of Bioscience, Aarhus University, Kalø, Grenåvej 14, 8410 Rønde, Denmark

**Keywords:** Aircraft, Airport, Bird strike, Geese, Population increase

## Abstract

We here review the collision risks posed by large-bodied, flocking geese to aircraft, exacerbated by recent major increases in northern hemisphere goose populations and air traffic volume. Mitigation of goose–aircraft strike risks requires knowledge of local goose movements, global goose population dynamics and ecology. Airports can minimise goose strikes by managing habitats within the airport property, applying deterrents to scare geese away and lethal control, but goose migration and movements at greater spatial scales present greater challenges. Habitat management outside of airports can locally reduce goose attractiveness of peripheral areas, but requires stakeholder involvement and coordination. Information on bird strike rates, individual goose movements and goose population dynamics is essential to understand how best to reduce the risk of goose strikes. Avian radar provides tactical information for mitigation measures and strategic data on local patterns of goose migration and habitat use. In the face of expanding air traffic, goose distributions and populations, these threats need to be integrated with other local, national and international stakeholder involvement to secure viable solutions to multiple conflicts.

## Introduction

Collisions between wildlife and aircraft (usually bird strikes) are increasing globally and present a disproportionate challenge around airports where aircraft are vulnerable during approach, landing and take-off (Dolbeer 2011). Geese constitute a particular hazard because of their flocking nature, large body size and attraction to extensive open landscapes of short managed grassland at airports. Although specific statistics are difficult to obtain, ducks and geese have been shown to constitute 20% of bird strike aviation accidents (Thorpe 2016), with geese globally responsible for at least 957 known reported bird strikes between 1983 and 1998, or 63.8 per year (Allan et al. 1999), and during 1990–2013 a total of 2015, or 84.0 per year, voluntarily reported strikes in the US alone (Dolbeer et al. 2014). Aircraft operators bear the brunt of the cost of damaging strikes, estimating to cost over US$ 1.2 thousand million annually throughout the world (Allan 2002). Single engine repairs may cost upwards of US$ 1 million without taking into account lost opportunity costs and service disruption from damage and downtime (Allan 2002). Beyond economic impacts, goose strikes present an extremely serious threat to human life when aircraft are damaged beyond their ability to sustain controlled flight. This has been demonstrated by events such as the crash landing of an United States Air Force E-3 Sentry at Elmendorf AFB in September 1995 that resulted in 24 fatalities and the forced landing of US Airways Flight 1549 in New York’s Hudson River in January 2009; both events were the result of engine ingestions of multiple Canada Geese *Branta canadensis* (Flight Safety Foundation 1996; Marra et al. 2009).

Airport operators are responsible under national and international legislation for mitigating the risks of bird strikes involving geese, and have used a variety of tools to achieve this aim. This is becoming particularly important given the rapid increases in some goose populations (see below) and the average 5.8% annual increase in global air traffic passengers between 2005 and 2016 (Statista 2016). Not surprisingly, in response to such universally operational challenges, there has been considerable development of international approaches and policy on the issue of bird strikes (see review in Buurma 2006) culminating in the development of safety standards ratified by the world community under the International Civil Aviation Organisation (IBSC 2006). In this analysis, we review some of the methods used to reduce goose presence in the onsite vicinity of airports, including habitat management, disturbance and lethal control. Geese exploiting habitats in the vicinity of an airport (but not within the direct jurisdiction of airport authorities) present greater challenges to management, requiring engagement with multiple stakeholders to influence land-use decisions and goose management at far greater spatial scales to reduce their prevalence. Analysis of the US Federal Aviation Administration bird strike data showed bird strike rates below 152 m above ground level have decreased since 1990, while those above that level have increased (Dolbeer 2011). The decrease in bird strikes below 152 m may be the response to effective implementation of measures onsite at airports and may suggest that there are growing issues associated with areas outside the jurisdiction of airports, especially within the landscape immediately surrounding airport ownership (Dolbeer 2011; Martin et al. 2011). For this reason, we here consider mitigation measures for dealing with geese both onsite and in areas outside airport boundaries.

The hazards presented to the aviation industry by geese are further exacerbated by rising goose populations in North America and Europe which elevate risks (see Fox and Madsen 1997; Dolbeer and Eschenfelder 2003; Fox and Leafloor 2017). It is therefore important to consider strategically how to manage geese beyond the local measures taken by airport operators and address the multiple problems posed by recent increases in goose abundance and range. As well as attempting to consider how best to tackle local problems posed by geese around airports, we also come with recommendations about how to establish long-term local, national and international involvement with other stakeholders to find common solutions to broader conflicts with geese. In particular, we recommend finding mechanisms to facilitate collaboration on research, experiences and data sharing, and the importance of a biological understanding of the behaviour, migration, distribution, ecology and population dynamics of the goose species causing issues.

## Understanding the nature of the problem

Problems associated with geese physically present onsite at an airport require very different solutions compared to those posed by geese traversing over the airspace of an airport. Long-distance migrant geese passing through during a brief annual migration window present a different level of risk and require different solutions to geese moving through the same airspace daily commuting between night time roosts and daily feeding areas, even when both lie offsite. It is therefore evident that a biological understanding of the behaviour, migration, distribution, ecology and dynamics of the goose population(s) concerned is fundamental for developing recommended mechanisms and best practice for reducing bird strike risk.

Migratory geese that breed in northern latitudes, but winter further south, pose a different set of challenges to local or resident birds that spend the greater balance of their annual cycle in one region for managers tasked with maintaining aviation safety. Migrant geese may pass through airspace infrequently, as they transit between breeding and wintering grounds; however, they may do so at altitudes where they are difficult to manage by ground personnel and in numbers that increase the likelihood of a damaging strike. Hence, the potential to manage the serious collision risks associated with migrating geese usually lies well outside of the area of influence of airport authorities. In such circumstances, all that can be done is to observe movements and alert pilots to these via the Automatic Terminal Information Service (ATIS) and/or direct communication with Air Traffic Controllers. When wintering grounds are juxtaposed with an airport, migrant geese can represent a persistent and significant risk. In contrast, local or resident birds may commute regularly between different elements in the landscape around the airport and represent a year-round hazard, but may be more predictable in their movements and more amenable to local management.

Copenhagen Airport at Kastrup (Denmark) experiences both migrant geese and resident geese. An example of a genuine migrant species is the barnacle geese *Branta leucopsis* that breed in the Russian Arctic and the Baltic, and winter around North Sea coasts. This population has shown exponential increase in recent years and exceeded 1.2 million individuals in 2015 (Fox and Madsen 1997). These geese pass through once annually in either direction in spring and autumn. These migration patterns are relatively regular in time and space (although mediated by local weather) and are therefore somewhat predictable in occurrence. In the case of locally based geese, the migratory barnacle geese contrast with those of the same species that nest on the island of Saltholm under the eastern approach to Copenhagen Airport (Christensen et al. 2015a). The local breeding birds also winter around North Sea coasts, but unlike their long-distance migratory counterparts, these geese remain in the vicinity of Copenhagen Airport throughout the summer (May–July, Christensen et al. 2015a). Telemetry studies of 10 individual females caught on Saltholm showed that these geese remained on Saltholm throughout much of their residency period, when they rarely flew above 20 m. The few tagged individuals that did not stay on the island to moult went to Sweden, whereas the rest travelled after they had completed the post-breeding moult. All geese departing Saltholm did so at low altitude (mean 25 m, maximum 240 m in August); only one individual ever traversed a runway approach (on a single occasion) and did so well below aircraft flight altitude. During the period that the geese spent in the region, they flew at an average altitude of 57 m (maximum 451 m) until their departure to winter quarters in October. Hence, despite their abundance and movements in the local proximity of the airport, these 10 individuals presented no hazard to air traffic. We should be extremely prudent about concluding that the movements of 10 tagged geese represent the typical movements of the 7000–10 000 barnacle geese present here in the late summer period. However, these data provide a useful insight into their movements and the risks that they present to bird strikes at the airport that will be important when undertaking an overall risk assessment of their presence and activity.

## Managing geese onsite

### Managing grass infields and removing water bodies to reduce goose habitat use

Most airports in Europe and North America maintain extensive areas of grassland between runways and taxiways to ensure pilot and general visibility, allow for emergency passage of aircraft straying from paved areas and enable rapid access for emergency vehicles to all areas (Washburn and Seamans 2004, 2013). However, very short-mown grassland attracts insectivorous flocking avian species (such as European starlings *Sturnus vulgaris*, gulls and shorebirds; Brough and Bridgman 1980). Furthermore, because geese forage on grass swards that have relatively high digestible protein content and are low in structural carbohydrates (Sedinger 1997; Fox et al. 2017), the shortest mown turf swards are highly attractive to them. Northern Hemisphere geese tend to forage on grazed or cut swards less than 15 cm high, so maintenance of tall (18–36 cm high) sward may render such areas less attractive to geese, especially if seedheads are allowed to form. However, this is not necessarily always the case (e.g. Cleary and Dolbeer 2005; De Vault et al. 2012; Washburn and Seamans 2004, 2013), because Seamans et al. (1999) observed no difference in goose use of swards that were 4–11 cm compared to 16–21 cm high. Furthermore, there are situations where it is not feasible to develop tall swards where short grassland remains a necessity between the taxiways and runways for visibility of lights and information signs, where tall grass is unattainable because of site conditions (for example, where restricted by climate, as in Iceland) or where complex interacting factors necessitate compromise to determine best management practices for mitigating multispecies bird strike risks (Blackwell et al. 2013a).

Attempts have been made to identify grass species that are unpalatable to geese (e.g. Conover 1991; Washburn et al. 2007; Washburn and Seamans 2013), while other efforts minimise the nutritional returns for geese; researchers in one study found that endophyte-infected turf-type tall fescue *Festuca arundinacea* was less attractive to captive geese compared to perennial rye-grass *Lolium perenne* and white clover *Trifolium repens* (Washburn et al. 2007). However, once a sward of a specific unpalatable grass species is established, it may require intensive management and reseeding to maintain in the face of recolonization by other grass species of higher palatability to geese (Washburn 2012). The use of nitrogen fertilisers can increase the nutritional composition of grass swards and make them more attractive to foraging geese (e.g. Riddington et al. 1997). Fertilisers should not be used on airfield swards unless to promote tall grass swards, and the use of nitrogen-containing compounds (e.g. urea) for de-icing paved surfaces likely increases the quality of grass habitats directly adjacent to the runways where such chemicals are applied (e.g. Gay et al. 1987).

Application of repellents (especially using methyl anthranilate, anthraquinone or their derivatives) has proved successful at small scales, although effective and relatively inexpensive in the short term, they often require frequent re-applications to maintain effectiveness (e.g. Mason and Clark 1995; van Liere et al. 2009; Ayers et al. 2010). Inoculation of grasses with endophytic fungi that produce alkaloids has been successful in reducing non-native Canada geese at New Zealand airports (Pennell and Rolston 2013). Copenhagen Airport is in the process of reseeding with a mixture of tall fescue *F. arundinacea* and perennial rye-grass *L. perenne* inoculated with a high content of such endophytes, but this approach has not been applied on a sufficiently large scale at airports to date to judge its effectiveness.

Wetlands habitats that attract geese for feeding or drinking (e.g. water bodies; Blackwell et al. 2013b) should be removed, if at all possible. If removal is not feasible, effort should be made to exclude geese, either by netting the wetland or establishing vegetation along the margin of the waterbody. Physical barriers along the water’s edge, such as a fence, may also prevent geese from using such open water. Guidance on implementation of such measures can be found in Conover and Kania (1991), Allan et al. (1995) and Smith et al. (1999).

### Active control

Despite all attempts to create a landscape that is as unattractive as possible to geese, in situations where geese land on airports, managers can employ a range of measures to disturb and displace geese away from the vicinity of arriving and departing aircraft. Methods that have been used include acoustic deterrents (e.g. gas cannons, blank cartridges, goose alarm calls), combined acoustic–visual deterrents (pyrotechnics), visual deterrents (lasers, most effective at lower light intensities, e.g. at night for dispersal from roosts) and deployment of potential predators (e.g. trained dogs and large birds of prey, see Hromádka 2013). The use of infrasound has been suggested but its effectiveness is not proven (Gilsdorf et al. 2002; Fidgen et al. 2005). Birds cannot hear in the ultrasound range, so this has been shown to be ineffective for avian scaring (Dooling 1982; Bomford and O’Brien 1990). Generally, any unfamiliar loud noise is highly effective at first application in displacing most birds; hence, the use of these and especially pyrotechnics (e.g. Aguilera et al. 1991) represents important tools exploited by airports against geese. However, regular and especially predictable use of any such techniques (even alarm calls and strong lasers) will result in habituation (although in one study, Canada geese showed no habituation over a period of 100 days; Whitford 2008). This necessitates that such methods are integrated into a cohesive strategy that incorporates their use in combination with lethal control (see below) and other techniques (Christensen et al. 2015b). The use of dogs to disperse birds on airports is widespread throughout the world and effective because dogs (especially dogs bred for hunting or shepherding) repeatedly target flocks specifically, are not subject to habituation and have been highly effective in displacing geese and in reducing frequency of airstrikes (e.g. Carter 2000; Castelli and Sleggs 2000; Froneman and Rooyen 2003; Allan 2006).

### Lethal control

Waterfowl are known to alter their distribution in response to hunting pressure (Fox and Madsen 1997; Madsen 1998), and the judicious use of lethal control can emulate a hunting environment around an airport. Lethal control may also serve to remove problem birds from the area. Many airports adhere to regulatory guidance by removing geese by lethal means onsite (mostly by shooting) as a last line of defence, although its use is highly dependent on location and operational constraints.

## Managing geese offsite

Management of habitats and the geese on the airfield alone is insufficient for alleviating the risk of a strike because geese will still pass through the airspace above the site and through the departure and approach corridors as they move between habitats in the vicinity of the airport. While the management of onsite geese can be intensive and targeted, and is largely at the discretion of the airport operator, the management of offsite geese requires significant collaboration with multiple stakeholders. These stakeholders may include local authorities and non-government organisations, as well as the private owners and occupiers of the land concerned. For this reason, airport authorities are often totally reliant on the cooperation of local owners and stakeholders to manage geese and goose habitats (even under formal management agreements) based on arguments put forward to justify them in relation to air safety concerns. The International Civil Aviation Organisation (ICAO) recommends a 13-km radius safety zone around an airport centre point for the basis of an effective wildlife management plan (ICAO 2012). Within such an area, airport authorities should also be consulted in the planning process to evaluate any new developments that might attract birds or their flight lines to the airport (ICAO 2012). Existing guidelines also provide advice on how to avoid attracting bird hazards within this area (ICAO 2002). In some cases, the actions necessary to achieve effective goose management may be in opposition to current land use or undesirable to the stakeholder responsible for the land in question.

In urban areas, open lawns and water bodies are publicly desirable amenities, and also increase the risk of attracting geese to the vicinity of the airport (Fox et al. 2013). Likewise, agricultural crop production can represent the mainstay of local farm economies but may increase the density of geese near an airport (Blackwell et al. 2009). In some cases, airports are able to extend the intensive management of geese outside their perimeter fences. For example, lesser snow geese *Chen caerulescens caerulescens* nesting in the Russian arctic migrate along the Pacific Coast in fall to wintering areas in British Columbia (Canada), Washington, Oregon and California (United States of America). Migration is prolonged from October to December, and thousands of birds remain on the Fraser River delta (British Columbia) all winter (Boyd 1995), in inter-tidal marshes directly adjacent to the Vancouver International Airport. These geese represent an offsite risk to aircraft using the airport. Under permit from federal authorities, the snow geese are actively controlled on the foreshore with pyrotechnics and judicious use of lethal control. However, in this case, agricultural habitats outside the airport operating area were proposed to contribute to mitigating the strike risk for aircraft by providing undisturbed offsite foraging areas as refuge for snow geese displaced from foreshore marsh areas. Federal officials had recognised that the exclusion of snow geese through hazing (i.e. persistent disturbance) would result in a functional exclusion of the birds from the foreshore marsh. Based on this, the Vancouver Airport Authority provided funds to establish fall-sown cereal crops as lures in an agricultural area 9 km to the south. This combination of mechanisms has been successful in maintaining goose wintering concentrations in agricultural fields well away from the airport (Bradbeer 2007). This experience underlines the results of studies of scaring from sensitive agricultural areas, namely scaring works most effectively for geese if developed in conjunction with the provision of safe haven refuges to which birds can be displaced and left to feed in peace (Hake et al. 2010; Kristiansen et al. 2005; Fox et al. 2017). Placement of sacrificial lure crops is critical in relation to loafing/roosting/drinking sites to ensure goose flight lines do not cross aircraft approach and departure corridors (Baxter and Robinson 2007).

Long-term management agreements with local land owners can be used as a mechanism for reducing the availability of attractive habitats around an airport. In the Netherlands, outside of Amsterdam, Schiphol International Airport is surrounded by thousands of acres of productive farmland. In former times, the post-harvest fields provided an abundance of crop residues, including cereals and vegetables, which attracted barnacle and greylag geese *Anser anser*, as well as other species of herbivorous waterfowl. To mitigate the risk of foraging geese in the vicinity of the airport, officials from Schiphol engaged their farming neighbours and entered into legal agreements that ensured all crop residues were tilled into the soil less than 48 h after harvest. In combination with population control, land management and use of technology in active control, the fields were attractive to geese only for a very few days, compared to previously when they attracted geese for several weeks (B. Straver pers. comm.). Monitoring of goose movements was undertaken using radar before and after the agreement was reached between Schiphol and local farmers, and these data showed a reduction in the number of goose movements over the airfield (van der Meide and Pieterse 2013).

Despite these examples, airports generally have exerted little influence on existing land use within airport safety zones outside the areas of their jurisdiction. Within such areas, influencing traditional agricultural practices through consultation with large numbers of private landowners and occupiers in extensive surrounding areas represents a major challenge. In contrast, airports are probably more successful in wielding influence through regional planning to avoid new developments that may affect bird occurrence (such as rubbish tips that might attract gulls). As lakes and wetlands are likely to attract geese and other waterfowl for foraging, loafing and night roosts, objection to new wetland restoration projects has been both appropriate and provided an administrative mechanism to avoid conflict in the vicinity of airports. In Denmark, where the recommendations from ICAO are implemented in the provisions of domestic legislation, airports have successfully objected to lake and wetland projects, when applied for these concerned areas within 13 km of the airports (cf. Christensen and Hounisen 2015).

Lethal control of locally resident geese may be an option for use offsite (for instance by supplementing oiling of eggs to destroy embryos by rounding up flightless geese in moult) when other mechanisms have failed, especially when dealing with resident populations of geese (as at John F. Kennedy International Airport with local Canada geese; Seamans et al. 2009). However, public acceptance of such actions may limit the degree to which lethal control can be adopted to manage populations (e.g. Colucy et al. 2001) confirming the care needed for sensitive and effective engagement with local communities to find amicable solutions. If lethal control is considered an option, the roundup of individuals must be extensive enough to impact the population in the vicinity of the airport. A study of neck-tagged Canada geese at Greensboro, North Carolina revealed that removing geese from a single site resulted in re-colonisation within only 27 days, underscoring the necessity of repeated removal of geese within an 8-km radius around an airport (Rutledge et al. 2015). The use of hunting to disturb geese and potentially regulate the population can offer a form of cost-neutral management option. Such a measure to manage geese outside of the airport perimeter may potentially enjoy public and hunter support. For example, special hunting seasons have been put in place in municipalities surrounding Copenhagen Airport in order to target geese that constitute a particular hazard and reduce their prevalence and density in the vicinity of the aerodrome. However, to the best of our knowledge, there has not been any strategic, coordinated use of hunting as a management tool to control geese in areas outside the airport, but within the 13-km radius, even though such an approach could potentially reduce the incidence of geese in core areas around the airport (cf. Christensen et al. 2015b).

## The need for improved data

The conflict between geese and aviation safety is a complex issue. Geese use a mosaic of habitats in and around airports, which necessitates research and monitoring to understand their spatial and temporal patterns of habitat use (e.g. areas used as roosts, loafing and freshwater drinking sites as well as places to obtain grit for digestion). The process of finding effective solutions to the conflicts these patterns pose to airport safety necessitates the active involvement of many diverse stakeholders. Local information about goose ecology is required to make informed management decisions and to assess and validate actions. Several potential sources of data relating to goose ecology in the vicinity may be available to airport authorities and their stakeholder partners, and mechanisms to generate such data can and need to be established. However, it is of fundamental importance to identify the specific data needs before initiating data collection.

**Fig. 1:**
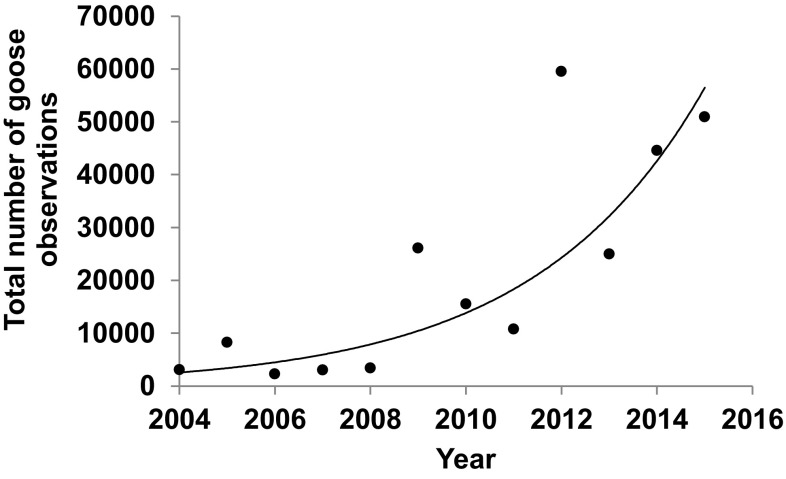
Increase in annual number of observations of geese of all species (mainly barnacle and greylag geese, but including small numbers of white-fronted and Canada geese) flying over or settling at Copenhagen Airport, 2004–2015. The rate of increase is equivalent to a 28% increase per annum (*r* ^2^ = 0.72, *P* = 0.0005) in response to constant effort in monitoring. Data courtesy of Copenhagen Airport

For example, at Copenhagen Airport, two species of geese constitute air safety risks, barnacle geese and greylag geese. As of 2015, nearly 4500 barnacle geese breed on the nearby island of Saltholm, but as was shown earlier, these birds constitute relatively little threat to the airport by virtue of their behaviour, dispersal and flight height. Their numbers are dwarfed by the passage of barnacle geese that breed throughout the Baltic Region and in Arctic Russia, which pass through airport airspace in spring and autumn to and from the breeding areas to winter quarters around North Sea coasts. This population has increased from 10 000 in the 1950s to well over 1.2 million currently, and during the period from 2004 to 2015 (Fox and Madsen 1997), the numbers of observations of geese of all species (i.e. mostly barnacle and greylag geese, but including small numbers of Canada geese and white-fronted geese *Anser albifrons*) observed over-flying or settling at Copenhagen Airport have increased exponentially (Fig. 1). Barnacle geese have only contributed to bird strikes since 2002, and the seasonal pattern (1 in March, 2 in May, 1 found dead in June, 1 in August and 4 in October) confirms that more collisions are associated with periods of peak migration than with the period when local breeding birds are present. Although these numbers are small, they support the hypothesis that it is migratory geese that are mostly being struck, but there is an urgent need for more data to confirm this pattern. Copenhagen Airport has initiated data collection on goose origins (i.e. local vs. migrant) using stable isotope analyses of tissues from corpses of geese shot on site and possible impacted geese. Stable isotope analysis of different feather tracts can identify the geographical and biological isoscapes in which those feathers were grown, which can greatly aid the identification of sub-populations to which these individuals originated and even reveal their recent diet and therefore habitat use (Inger and Bearhop 2008). As data accumulates over time, it will become more evident as to whether it is local or migrant geese that pose a risk to air safety and how the balance of local/migrant geese change over time. Such information will be vital to wildlife managers to use as guidance when developing new strategies towards geese. Moreover, data from stable isotope analysis in combination with observational and radar studies would strengthen our understanding underlying the patterns and trends and may even enable confirmation of the relative risks posed by (and the actual levels of collision caused by) these two populations now and in the future. Greylag geese are also composed of relatively small (but increasing) numbers of local breeders, but which are supplemented by 40 000 moult migrant geese that aggregate from throughout the Baltic Region to replace their flight feathers on Saltholm in May and June. Although more difficult to determine, it seems likely that the greatest risk to flight safety is posed by arriving and dispersing moulting geese. However, since 2002, greylag goose bird strike incidents have been reported in February (2) and March (1), when migrant greylag geese were likely involved and July (1) when a local bird was likely involved.

These cases identify the vital importance of understanding the precise nature of the structure of the goose populations which potentially pose the local threat to airport safety, not just in terms of the species involved but also to which sub-populations they may belong in order to provide an adequate basis for the development of management solutions. Given such an understanding, it is clearly important to have basic data on the annual changes in abundance of both local sub-populations and the overall flyway populations to which these belong in order to understand the degree to which the airstrike risk is changing through time. Additional knowledge on annual reproductive success and survival would support the development of population models that would help guide adaptive management options, for instance, setting dynamic targets for lethal control at levels necessary to halt or reduce population size (e.g. Johnson and Williams 1999).

The use of individually marked birds can play a major role in assessing how geese move throughout the annual cycle and can serve to identify particularly hazardous sub-populations (e.g. Seamans et al. 2009). The increasing use of telemetry devices to provide spatial data in three dimensions has greatly advanced our ability to provide fine-grained information on movements of individual birds. Data from marked individuals can be used to inform external stakeholders of how geese using their sites impact air safety, making them more willing to adopt mitigation strategies. Neck bands were used to mark established free-flying Canada geese in the UK, and the re-sighting data informed managers of the need to remove cereal crops adjacent to the airport, an action which eliminated Canada geese from over-flying that particular airport (Baxter and Robinson 2007). This scientific understanding of the ecology of the geese and their behavioural patterns enabled a substantial reduction in the levels of management effort and eliminated bird strike with geese at the site. Airport authorities should create relationships with academic or relevant government institutions in order to exploit the necessary expertise to conduct a study of individually marked geese and their habitat use and incorporate this knowledge into the development of mitigation actions.

Compilation of strike data over time, collected by airport authorities and federal transportation regulators, provides measures of the risk associated with particular species. Detailed strike data that includes the prevalence of damage due to bird strikes serve to quantify when and where hazardous incidents occur and thus direct management to reduce the hazard. Like population data, long-term datasets serve as reference points for assessing trends over time and their relation to environmental and anthropogenic factors to support development of options to reduce risk. Comparisons of the incident frequencies can serve to measure efficacy of mitigation measures through time, as was done at Schiphol International Airport to measure the impact of offsite agricultural crop residue management (Van der Meide and Pieterse 2013). Information about the distribution of birds in the air and on the ground can be used to reduce the risk of bird strikes and their impact on operations as well as in and around airports. Shamoun-Baranes et al. (2008) used methods that predicted bird densities across The Netherlands and in Alaska to develop bird avoidance models for aviation. The models integrated data and expert knowledge on avian distributions and migratory behaviour to generate GIS-enabled Web-based hazard maps. Both systems are in operational use for flight planning and for airport and airport vicinity management.

Digital radar provides tracking of bird movements (especially large, flocking birds such as geese, which provide a robust radar reflection; Nohara et al. 2011) and provides spatial information over a range of distances and heights around airports, along with the associated risk of a strike occurring. These radar data can give immediate tactical information about the threat of bird strikes with target flocks, often detected at long distances from the airport (e.g. Nohara et al. 2011; although only out to 4 nautical miles in the case of Gerringer et al. 2016). Detection at long distance is especially important prior to entering airport airspace and presenting a threat to air traffic because it provides time to mount appropriate responses. Such intervening avian traffic can then be exposed to a range of mitigation measures (e.g. Pieterse 2014), including management of the flocks using active control. In situations where strong relationships exist between airport wildlife managers and Air Traffic Controllers, avian radar can be used to manage air traffic or alert air crews of hazardous geese in real time.

Avian radar can also provide long-term strategic data that can illuminate patterns of habitat use, phenology and frequency of high hazard birds crossing active airspace, although ground clutter generally prevents radar picking up geese on the ground. Over time, collection and collation of such data can be used to identify long-term trends, thereby predicting goose movements, especially the periods of peak migration, as well chart the build-up of numbers in different parts of the airport where they roost or feed to optimise targeting of these areas for most effective active control. Avian radar can also identify the development of offsite hazards, providing leverage to encourage stakeholders outside of the perimeter fence to implement appropriate measures.

## The way forward: From information to collaboration

While this review has shown many effective cases of local management being used to resolve conflicts between increasing goose populations and a concomitant increase in bird strikes, there is a much wider issue associated with major changes in goose abundance at the flyway level. The specific link between increases in goose flyway abundance, volumes of air traffic and the specific rate/cost of goose collisions has not been established, but at Copenhagen Airport barnacle goose strikes have occurred since 2002, when the population broke 500 000 individuals and that population now exceeds 1.2 million individuals (Fox and Madsen 2017).

Hence, while airport authorities can do all they can to dissuade geese from settling or flying close to airport airspace and to alert flight traffic to movements through early warning avian radar systems, the greater problem of managing overall goose population size requires concerted actions and a collaborative international approach. For this reason, national civil aviation authorities (e.g. The Civil Aviation Authority in the UK) and the airport managers need to join with consortia of other stakeholders in collaborating towards attaining the goal of maintaining the highest levels of regional aviation safety. This can be achieved by joining with much larger initiatives that contribute to solving the problems posed by increasing goose populations. Such a major initiative requires a scale-dependent set of mechanisms, starting at finding strategic solutions to conflict at the flyway level, but focussing ultimately for delivering solutions at the site level. Such a process requires an openness and willingness of all parties to work together with mechanisms to gather appropriate data sources and establish a shared understanding of the available data, a process that has been described by Tombre et al. (2013) in relation to agricultural conflicts with geese. Stakeholders are gathered to review scientific data and develop agreement on the data platform used, including population data and extent of risk or conflict. This important step would allow detailed data on goose ecology to be used to inform management actions. Federal authorities should be involved at the regional level so that population targets can be adopted into national species management strategies. Federal authorities will likely have access to the most comprehensive population data, allowing them to help regional stakeholder groups make informed decisions about agreed goose population targets. In the case of migratory geese, involvement of federal authorities can be the vehicle for management of regional goose issues at the flyway level with international partners.

There remain considerable improvements to be made at the regional level, where collaboration is required to engage regional stakeholders and raise awareness of the risk that geese pose to aircraft operations, including the worst-case consequences of an incident. This awareness, built upon an agreed acceptance of the current data, can be used to build a framework of cooperative management that helps to address goose issues on lands within the vicinity of the airports that are outside its jurisdiction.

Stakeholders involved should include local governments with jurisdiction over public green spaces, agricultural managers (if present in the vicinity of the airport), other private landowners with goose habitat on their properties and environmental organisations. Efforts should be made to discuss the differing interests of each stakeholder group to reach understandings and to unite interests (Tombre et al. 2013; Madsen et al. 2017). Synergies between different stakeholders should be identified, as a variety of conflicts with a specific goose population may exist for multiple stakeholders (e.g. a rising goose population that impacts aviation safety may also be responsible for increases in agricultural crop depredation). A united approach to goose management can help stakeholders achieve consensus on meaningful mitigation measures and population targets that are comprehensive of air safety requirements and other goose conflicts. Stakeholder collaboration can also facilitate public awareness, particularly if stakeholders can standardise the messaging they wish to share with the public. The importance of maintaining collaborative relationships with a wide range of stakeholders is heightened by the plasticity with which geese can adapt to using new habitats, especially within semi-urban and urban environments.

Geese represent an adaptable suite of species that have shown themselves well able to thrive within the mosaic of post-industrial human-modified landscapes, especially agricultural landscape and amenity grasslands. The risk of goose strikes to aviation safety has been an unintentional consequence of goose management efforts as well as the major expansion in the air transport industry over the past century. The long-distance migratory nature of many goose populations, both regionally and internationally, necessitates the collaboration of stakeholders at multiple levels (see Madsen et al. 2017). The ability to mitigate the risk of goose–aircraft collisions will be increased if regional management of wild geese is linked to international strategies. Stakeholders representing national interests (e.g. federal authorities responsible for managing goose populations) and scientists studying geese across their geographical range are the most likely bridges between regional and international stakeholder interests, but it remains essential that the lead in managing the risk of goose strikes is taken by the authorities responsible for aviation safety.
